# Recombinant Newcastle Disease Virus (NDV) Expressing Sigma C Protein of Avian Reovirus (ARV) Protects against Both ARV and NDV in Chickens

**DOI:** 10.3390/pathogens8030145

**Published:** 2019-09-10

**Authors:** Deep Prakash Saikia, Kalpana Yadav, Dinesh C. Pathak, Narayan Ramamurthy, Ajai Lawrence D’Silva, Asok Kumar Marriappan, Saravanan Ramakrishnan, Vikram N. Vakharia, Madhan Mohan Chellappa, Sohini Dey

**Affiliations:** 1 Recombinant DNA Lab, Division of Veterinary Biotechnology, Indian Veterinary Research Institute, Izatnagar, Bareilly 243 122, India; 2 Avian Diseases Section, Division of Pathology, Indian Veterinary Research Institute, Izatnagar, Bareilly 243 122, India; 3 Immunology Section, Indian Veterinary Research Institute, Izatnagar, Bareilly 243 122, India; 4 Institute of Marine & Environmental Technology, University of Maryland Baltimore County, Baltimore, MD 21202, USA

**Keywords:** newcastle disease virus, avian reovirus-σC protein, bivalent vaccine candidate, humoral and cell mediated immune responses, protection efficacy

## Abstract

Newcastle disease (ND) and avian reovirus (ARV) infections are a serious threat to the poultry industry, which causes heavy economic losses. The mesogenic NDV strain R2B is commonly used as a booster vaccine in many Asian countries to control the disease. In this seminal work, a recombinant NDV strain R2B expressing the sigma C (σC) gene of ARV (rNDV-R2B-σC) was generated by reverse genetics, characterized in vitro and tested as a bivalent vaccine candidate in chickens. The recombinant rNDV-R2B-σC virus was attenuated as compared to the parent rNDV-R2B virus as revealed by standard pathogenicity assays. The generated vaccine candidate, rNDV-R2B-σC, could induce both humoral and cell mediated immune responses in birds and gave complete protection against virulent NDV and ARV challenges. Post-challenge virus shedding analysis revealed a drastic reduction in NDV shed, as compared to unvaccinated birds.

## 1. Introduction

Viral arthritis/tenosynovitis is an economically important, widespread disease that affects the broiler breeders and flocks [1,2]. The disease is caused by avian reovirus (ARV), belonging to the *Orthoreovirus* genus of the family *Reovirdae*. Besides arthritis/tenosynovitis, the virus shows other clinical signs, causing gastroenteritis, hepatitis, myocarditis, malnutrition syndrome, chronic respiratory disease and affecting the central nervous system [3,4,5]. 

Avian reovirus is an icosahedral, non-enveloped virus consisting of ten segmented, double-stranded RNA genome. Based on their electrophoretic mobility, the ten segments comprise three each of L-class (L1, L2 and L3), M-class (M1, M2 and M3) segments, and four S-class segments (S1, S2, S3 and S4). The genome encodes eight structural proteins (λA, λB, λC, μA, μB, σA, σB and σC) that are found in mature viruses and four non-structural proteins (μNS, P10, P17 and σNS) are only induced in the infected cells [6]. The sigma C (σC) is a homotrimer protein located on the outer capsid and is responsible for cell attachment and induction of neutralizing antibodies. The mutation in the S1 segment encoding σC is responsible for segment rearrangement resulting in genetic diversity among reovirus [7]. 

The ARVs are relatively resistant outside the host and could be transmitted vertically without the detectable seroconversion and remain undetectable in cloacal swabs. The main approach to control the disease is by using live and inactivated vaccines, which provide maternally derived antibodies (MDA) to the progeny for preventing vertical transmission and protecting the breeder flocks from infection. Vaccination of the layer birds with a live vaccine in their early age, followed by vaccination with an inactivated vaccine at 6-weeks of age and again before the onset of egg-laying helps in the induction of high levels of MDA [2]. However, the currently available vaccines do not provide sufficient protection [8]. The genetic and antigenic variations in field isolates may be the reason for failure. Several companies are now developing autogenous vaccines generated from the locally prevalent virus isolates but are deficient in producing memory B and T cell responses, mucosal immunity, and long duration immunity. Attenuation of viruses can be a long-term process and costly. Moreover, live reovirus vaccines in hens are not efficacious in boosting antibodies and may result in economic losses in hens and their progeny [9], when compared to inactivated oil emulsion vaccines prior to the start of egg production, which may be due to poor intestinal immunity (IgA) developed following vaccination at a very young age [10]. Inactivated vaccine (S1133 strain) given to layer birds can reduce the occurrence of lesions in the hock joints of chicks, following a challenge at day old age [11]. Inactivated ARV vaccines are generally given in combination with other killed vaccines against Newcastle disease and egg drop syndrome 1976 (EDS’76) to breeder birds. Generally, the inactivated vaccines work more effectively if it is preceded by vaccination using a live vaccine [12,13]. Hence, strategies to generate newer vaccines are needed to provide a broader spectrum of protection. Viral vectored vaccines could be an alternate approach to control ARV infection. A number of attenuated poultry vaccine viruses, such as Newcastle disease virus (NDV) [14,15,16,17], fowl pox virus (FPV) [18,19,20], adenovirus [21], herpesvirus of turkeys (HVT) [22,23], infectious laryngotracheitis virus (ILTV) [24,25] and Marek’s disease virus (MDV) [26,27,28] have been genetically modified in order to use them as vaccine vectors for developing combinations of live attenuated vaccines for controlling the diseases of poultry. Among these viruses, NDV is an attractive vaccine vector for both human and animal diseases. The genome of NDV can be easily manipulated using reverse genetics technology [29,30,31,32] for developing live attenuated and bivalent vaccines against economically important poultry diseases. The advantages of recombinant viral vaccine over conventional vaccine would be the induction of both humoral and cell mediated immune responses and, at the same time, it will not create any variant vaccines.

Newcastle disease (ND) is another economically important poultry disease causing huge losses to the poultry industry. ND is caused by Newcastle disease virus (NDV) belonging to genus avian *orthoavulavirus 1* of family *Paramyxoviridae*. NDV strain R2B is used in India as a booster vaccination of older birds (≥6 weeks of age). The R2B strain was originated while passaging Indian field isolates in embryonated chicken eggs for 19 passages at the Indian Veterinary Research Institute, India [33]. The complete genotypic characterization of R2B has given new insights into the epidemiology of NDV and revealed that it is a mesogenic strain, with a genome size of 15,186 nucleotides, having a polybasic amino acid motif at its fusion protein cleavage site (FPCS), phenylalanine at amino acid position 117, and belonging to genotype II of class II cluster of NDV phylogeny [34]. India is an endemic country for both NDV and ARV, the present study was conducted to develop a live recombinant virus wherein, the R2B strain of NDV could express the σC protein of ARV and the generated bivalent vaccine candidate could be administered to 6–8 weeks old birds in order to induce protection against both NDV and ARV. The bivalent vaccine can be used as a booster vaccination of breeding stock for protection against NDV and ARV. To the best of our knowledge, a pioneer work has been conducted on Indian NDV R2B strain expressing σC gene of virulent ARV isolate to protect the chickens against both virulent NDV and ARV challenges.

## 2. Results

### 2.1. Generation of Recombinant NDV-R2B Expressing the σC Protein of ARV

**Figure 1:**
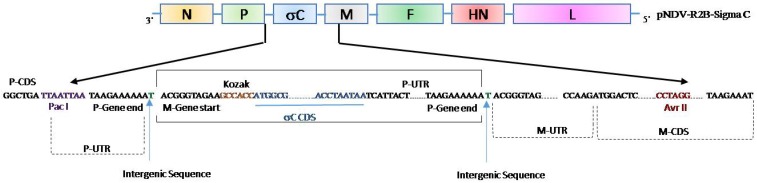
Generation of a full-length infectious clone of NDV-R2B containing the σC gene of avian reovirus. The σC gene cassette containing the NDV gene start and gene end signals was engineered to be incorporated between the P and M genes of NDV-R2B genome flanked by the restriction enzymes *Pac*I and *Avr*II. A Kozak sequence was incorporated preceding the σC gene coding sequence and one extra stop codon was artificially introduced at the end of σC coding sequence to maintain the “rule of six”. P-UTR and M-UTR contains the regulatory sequences of NDV, whereas P-CDS and M-CDS represent the complete ORF of P and M genes, respectively.

The σC gene cassette of 1.7 kb (obtained by overlap PCR), containing the σC gene flanked by start and stop signals of NDV, was inserted between the non-coding regions of P and M genes of the viral genome to obtain plasmid pNDV-R2B-σC (Figure 1). The recombinant NDV strain R2B expressing the σC gene of ARV (rNDV-R2B-σC) was rescued by transfecting the Vero cells with the full-length pNDV-R2B-σC and other support plasmids. Sequence data of the rescued virus confirmed that the junctions of the NDV, for the σC gene in the transcription cassette, were intact confirming the recombinant nature of the virus generated by reverse genetics. The genetic stability of the recombinant virus was confirmed by sequencing the viral RNA obtained after 10 serial passages in embryonated chicken eggs. The sequencing results indicated the integrity of the σC gene, and its expression was preserved even after 10 serial egg passages. 

### 2.2. Biological and Molecular Characterization of the Recombinant Virus rNDV-R2B-σC 

**Figure 2:**
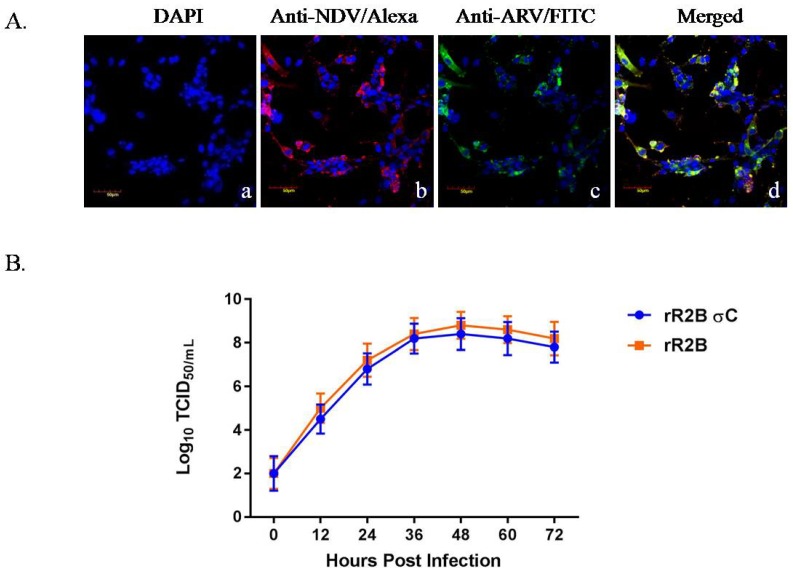
(**A**) immunofluorescence analysis of rNDV-R2B-σC virus expressing sigma C protein ×60. (**a**) DAPI stained cells; (**b**) cells expressing NDV proteins (Anti-NDV/Alexa); (**c**) cells expressing σC protein (Anti-ARV/FITC); (**d**) merged; (**B**) multistep growth kinetics of recombinant virus rNDV-R2B-σC in Vero cells. Monolayer of Vero cells were infected with rNDV-R2B-σC virus at MOI of 0.01 and culture supernatant was collected at every 12 h interval until 72 h, and viral titres were determined by limiting dilution assay and calculated as TCID_50_ by the Reed and Muench method.

Expression of σC protein in the recombinant virus was determined by immunofluorescence assays using hyperimmune serum against NDV and anti-reovirus antibody. When cells infected with the recombinant virus were examined under the confocal fluorescent microscope, a bright green fluorescence was observed, which indicated the expression of σC protein, and a red fluorescence signal was detected in the infected cells, which confirmed the presence of NDV (Figure 2A).

### 2.3. Growth Kinetics of the Recombinant Virus

The replication kinetics of the recombinant rNDV-R2B-σC and the rNDV-R2B viruses were determined by multi-cycle growth kinetics study in Vero cells. At indicated time points, the supernatants from infected cells were collected and titrated in Vero cells. Figure 2B depicts the growth curves of rNDV-R2B-σC and the rNDV-R2B viruses. There was a slight reduction in titre of rNDV-R2B-σC, as compared to the rNDV-R2B at all the time points studied. The maximum titre of rNDV-R2B and rNDV-R2B-σC were 10^8.8^ and 10^8.4^ TCID_50_/mL, respectively, at 48 h post infections (hpi).

### 2.4. Pathogenicity Analysis of the Recombinant Viruses

Mean death time (MDT) and the intracerebral pathogenicity index (ICPI) scores of the rNDV-R2B virus were 63 h and 1.45 and for the rNDV-R2B-σC virus were 72 h and 1.2, respectively. Both the MDT and ICPI assays indicated the rescued recombinant virus to be mesogenic in nature, with a slight reduction in virulence as compared to the parent recombinant virus.

### 2.5. Evaluation of the Protective Efficacy of Various Recombinant Viruses against Virulent ARV and NDV Challenges in Chickens

**Figure 3:**
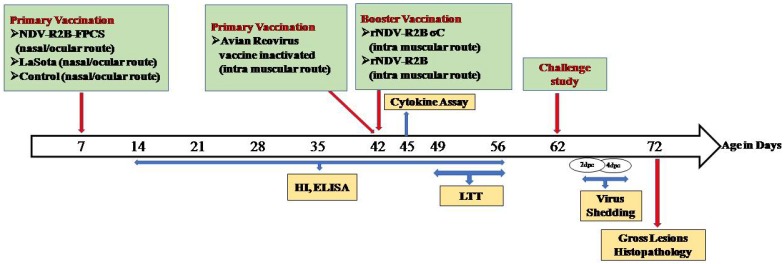
Schematic diagram showing the experimental design. NDV: Newcastle diseases virus, ELISA: enzyme-linked immunosorbent assay, HI: haemagglutination inhibition test, LTT: lymphocyte transformation test, dpc: days post challenge.

The immunogenicity and protective efficacy of the recombinant viruses generated in the laboratory, namely, rNDV-R2B-FPCS [35], rNDV-R2B [16] and rNDV-R2B-σC, were evaluated in specific pathogen free (SPF) chickens. The recombinant virus rNDV-R2B-FPCS and commercial LaSota strain virus were used as primary vaccine candidates and rNDV-R2B and rNDV-R2B-σC were used as booster candidates, as summarized in Figure 3 and Table 1. 

#### 2.5.1. Assessment of Humoral Immune Response

**Figure 4:**
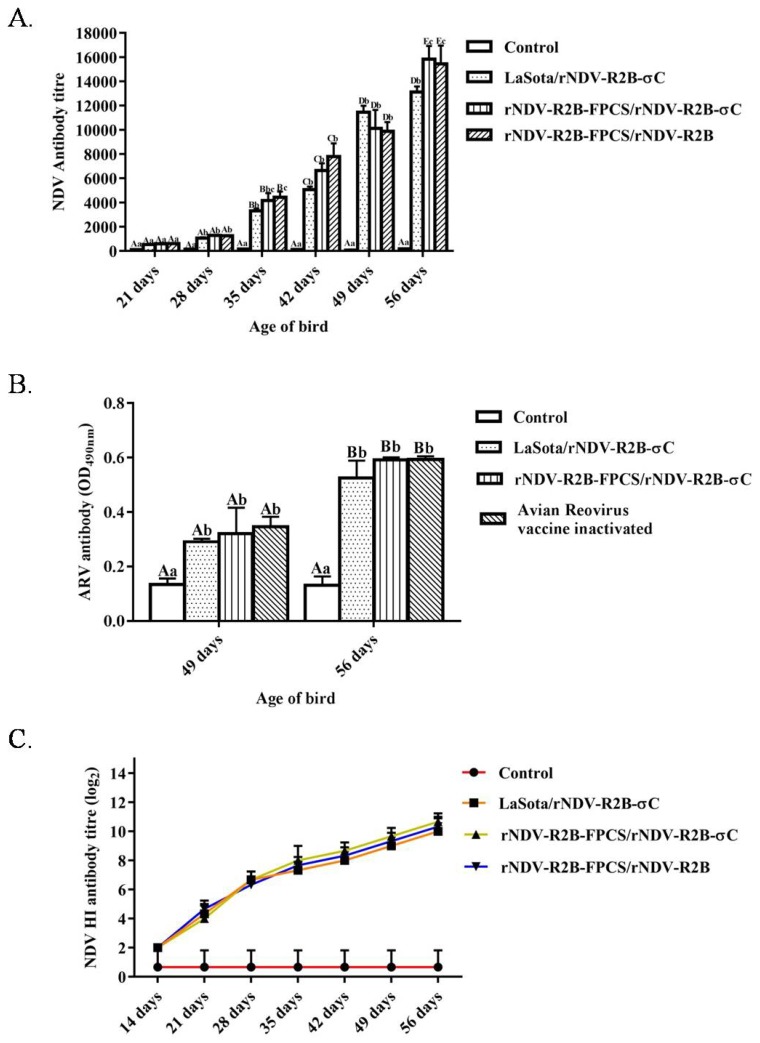
Assessment of NDV and ARV specific serum antibodies in experimental chickens by ELISA and HI. (**A**) birds of three groups were immunized at seven days of age with rNDV-R2B-FPCS and live LaSota vaccine as a primary vaccine. The control group birds were injected with phosphate buffered saline (PBS). Booster dose was given at 42 days of age with rNDV-R2B-σC and rNDV-R2B viruses to the corresponding groups and the control group was again injected with PBS. Serum samples were collected from the immunized and control group of birds at regular intervals and tested for NDV specific antibodies. The antibody titres higher than 200 was considered positive for NDV specific antibody; (**B**) one group was vaccinated with ARV inactivated vaccine at 6^th^ week of age. Serum samples were collected from the immunized and control group of birds at regular intervals and tested for anti- σC antibody. The O.D. value > 0.1779 (mean O.D. of the control birds + 3 S.D.) were considered positive for ARV antibodies. Bars (mean ± SE) indicate the representative data of a single experiment. Data with different capital letters superscript indicates the time effect (*p* < 0.01) and small letters superscript indicates the treatment effect (*p* < 0.05); (**C**) assessment of NDV specific serum antibodies in response to vaccination as determined by an HI test. Serum samples were collected at 14,21,28,35,42,49 and 56 days of age from all the birds. All HI titres were expressed as mean reciprocal log2 titre + SEM (standard error of the mean) (n = 10). Statistical differences were calculated by one-way ANOVA with *p* < 0.01 and Waller–Duncan as a post hoc test.

Humoral immunity induced in the experimental birds in response to vaccination was determined by ELISA and HI test for NDV specific antibodies at 14, 21, 28, 35, 42, 49 and 56 days of their age. Similarly, antibodies generated against the ARV σC antigen were determined by ELISA at 49 and 56 days of age following the booster vaccination at 42 days of their age. As a response to vaccination, all of the birds developed NDV specific antibodies with time. In ELISA, a significant difference was observed from 21 days in the level of NDV specific antibodies in the vaccinated groups as compared to the unvaccinated control group (*p* < 0.01). In ELISA, a significant difference was observed in the level of NDV specific antibodies in the birds primed with rNDV-R2B-FPCS vaccine candidate until 42 days of their age. Furthermore, the birds primed with altered FPCS, namely rNDV-R2B-FPCS, and boosted with rNDV-R2B-σC and rNDV-R2B vaccine candidates showed the highest antibody titres at 56 days of age without any significant difference between them (Figure 4A). 

The level of anti-σC antibodies was determined at 49 and 56 days of age as mean absorbance at 490 nm (OD_490nm_) and was compared to that of the cut-off value 0.1779 (mean OD value ± 3SD). The serum samples above the cut-off value were considered as positive. The ARV specific antibody titres increased with the time point of the experiment and a significant difference (*p* < 0.01) was observed between the vaccinated and unvaccinated birds. However, the rNDV-R2B-σC virus could induce ARV specific antibodies as effective as the commercially available inactivated vaccine at 49 and 56 days of their age (Figure 4B). 

The level of haemagglutinating antibodies in an HI test showed similar trends with ELISA titres during all the time points tested suggesting that rNDV-R2B-FPCS and rNDV-R2B-σC are as effective as the LaSota strain at inducing HI antibodies (Figure 4C). 

#### 2.5.2. Assessment of Cell Mediated Immune Response

The cell mediated immune response was determined by antigen specific lymphocyte proliferation as measured by lymphocyte transformation test (LTT) and cytokine gene expression analysis of activated peripheral blood mononuclear cells (PBMCs).

**Figure 5:**
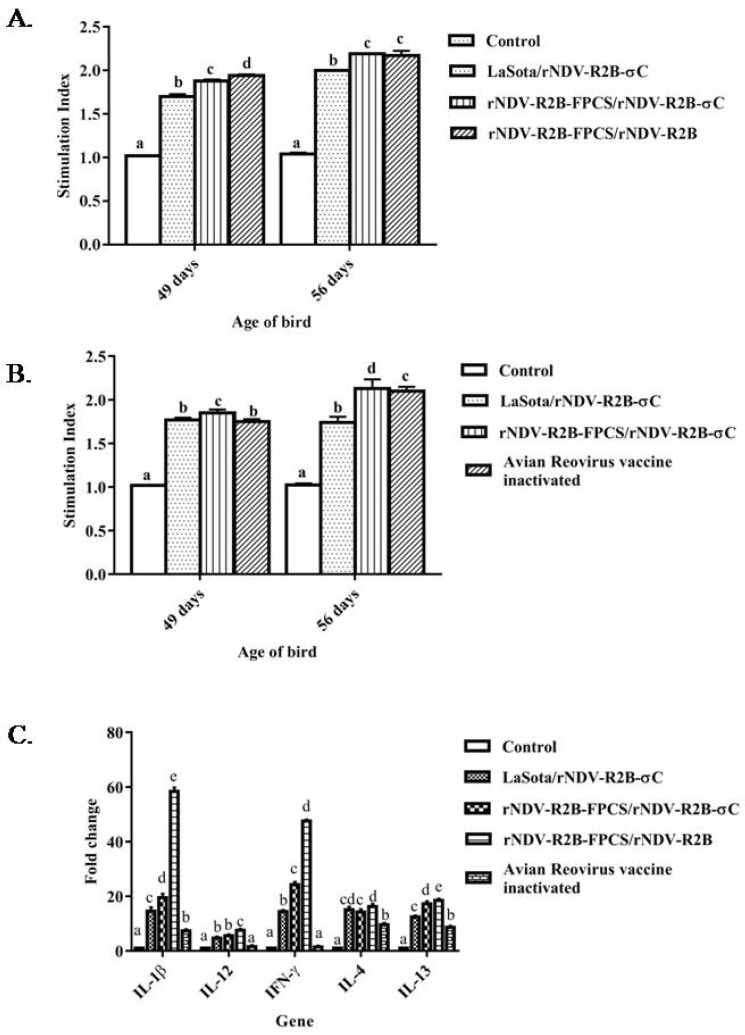
Assessment of antigen specific lymphocyte proliferative response in chickens at 49 and 56 days of age. Chicken PBMCs from immunized and control groups (n = 6) were stimulated with (**A**) NDV and (**B**) recombinant σC antigen expressed in *E. coli.* Bars (mean ± SE) indicate the representative data of a single experiment. Lymphocyte proliferative response was measured and expressed as stimulation index. Level not connected by same letter is significantly different (*p* < 0.05). (**C**) mRNA expression of different cytokine genes in response to vaccination as assessed by quantitative real time PCR and normalized to β-actin gene. Relative expression was determined by using the 2−^ΔΔCt^ method and relative fold change of each cytokine expression between vaccinated and control groups are presented. All of the data are represented as mean value ± standard errors. Data with different small letter superscript indicates the treatment effect (*p* < 0.05).

The antigen specific lymphocyte proliferation was determined at 49- and 56-days age of the birds. There was a significantly higher lymphocyte proliferation in the vaccinated birds as compared to that of control birds against NDV and ARV σC specific antigens (*p* < 0.05). At 49 and 56 days of age, there was a significantly higher proliferation against NDV antigen in the group vaccinated with rNDV-R2B-FPCS/rNDV-R2B viruses as compared to other groups (Figure 5A). The NDV antigen specific proliferation at 56 days of age was comparable between rNDV-R2B-FPCS/rNDV-R2B-σC and rNDV-R2B-FPCS/rNDV-R2B vaccinated groups. Similarly, the ARV σC antigen specific proliferative response was observed in the birds at 49 and 56 days of their age. Significant differences in the proliferative responses were seen among the groups at 56 days of age and the highest response was observed in the birds vaccinated with rNDV-R2B-FPCS/rNDV-R2B-σC viruses followed by the birds immunized with the ARV inactivated vaccine (Figure 5B).

The messenger RNA (mRNA) expression pattern of five different cytokine genes viz., IL-1β, IL-12, IFN-γ, IL-4 and IL-13 were determined in the birds at 45 days of age by quantitative real-time PCR. There was a significant difference in the expression level of IL-1β among the vaccinated birds and the highest level was observed in rNDV-R2B-FPCS/rNDV-R2B virus groups, followed by rNDV-R2B-FPCS/rNDV-R2B-σC, LaSota/rNDV-R2B-σC groups and the birds immunized with ARV inactivated vaccine. Similarly, the highest level of IL-12 expression was detected in birds vaccinated with rNDV-R2B-FPCS/rNDV-R2B virus group and there was no significant difference in the expression of IL-12 between rNDV-R2B-FPCS/rNDV-R2B-σC and LaSota/rNDV-R2B-σC groups. Moreover, there was no significant difference in IL-12 expression levels between the control and the commercial ARV inactivated vaccine groups. The highest level of IFN-γ expression was observed in the birds vaccinated with rNDV-R2B-FPCS/rNDV-R2B groups, followed by the birds in the rNDV-R2B-FPCS/rNDV-R2B-σC vaccine groups. Similarly, there was no significant difference in the IFN-γ expression level between the control and the commercial ARV inactivated vaccine groups. There was a significant difference in the IL-4 expression levels, the highest being observed in rNDV-R2B-FPCS/rNDV-R2B groups, followed by rNDV-R2B-FPCS/rNDV-R2B-σC and LaSota/rNDV-R2B-σC groups, with the lowest in the commercial ARV inactivated vaccine group. The level of expression of the cytokine IL-13 was also found to be the highest in rNDV-R2B-FPCS/rNDV-R2B group, followed by the rNDV-R2B-FPCS/rNDV-R2B-σC group of birds (Figure 5C).

### 2.6. Protection Studies on Experimental Specific Pathogen Free Chickens

**Figure 6:**
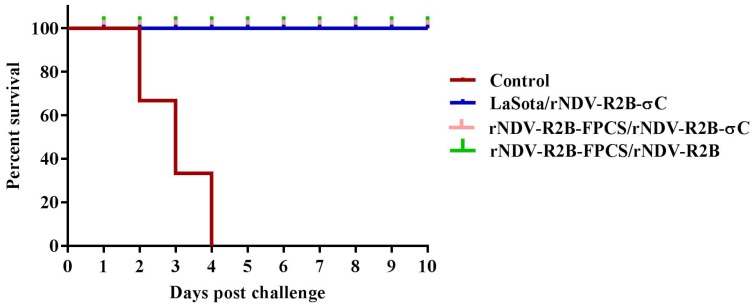
Survival curve for birds immunized with recombinant and vaccine viruses. Birds were challenged with 10^5^ ELD_50_/mL virulent NDV at 62 days of their age and survivability was assessed up to 10 dpc. All of the control birds died by 4 dpc.

All the birds immunized with the recombinant viruses rNDV-R2B-FPCS/rNDV-R2B-σC, rNDV-R2B-FPCS/rNDV-R2B, live vaccine LaSota and ARV inactivated vaccine were completely protected when challenged respectively against virulent NDV and ARV at 62 days of their age, without showing any clinical signs against these diseases. All the control birds exhibited typical NDV disease symptoms within four days post-challenge (dpc) with the virulent NDV (Figure 6). The unvaccinated control birds that were challenged with virulent ARV survived; however, clinical symptoms typical of ARV infection were observed. 

### 2.7. Post Challenge Analysis of Virus Shedding

**Figure 7:**
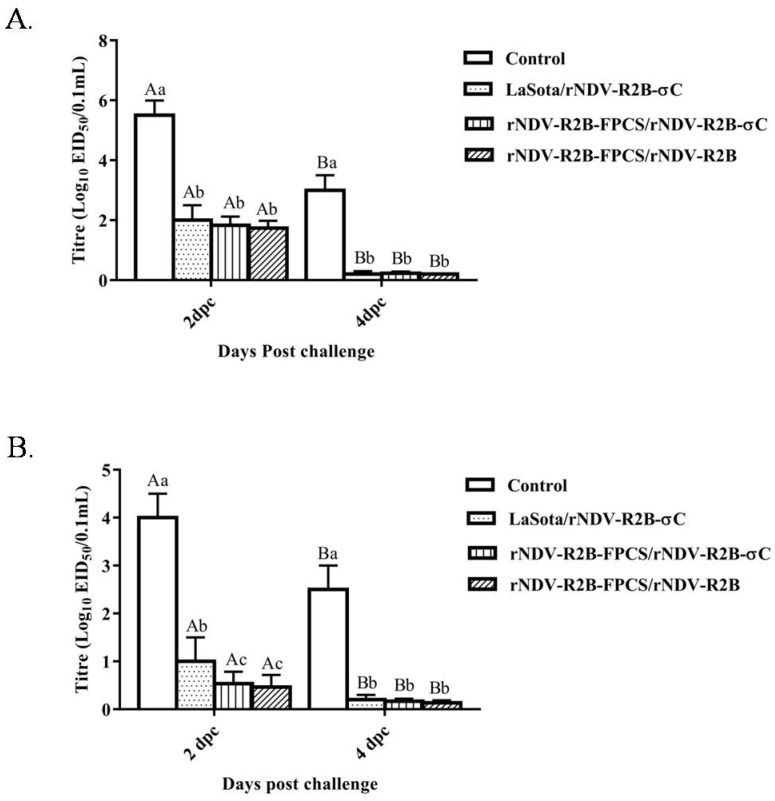
Reduction of virus shed due to vaccination in comparison to unvaccinated control group. (**A**) oropharyngeal and (**B**) cloacal swabs were collected on 2 and 4 dpc from all the birds for virus isolation. By day 4, all the control birds died due to an NDV challenge, whereas the viral shed from all the vaccinated groups reduced drastically by 4 dpc as compared to the control. Data with different small letter superscript indicates the treatment effect (*p* < 0.01) and capital letter superscript indicates the time effect (*p* < 0.01).

Oropharyngeal and cloacal swabs were collected aseptically for analysis of NDV shedding from the birds on 2 and 4 dpc. All the oral and cloacal swabs collected from control and vaccinated birds were found to be positive for the presence of NDV on 2 dpc. However, there was a significant reduction in the viral shed at 4 dpc (both oral and cloacal) in the birds vaccinated with LaSota/rNDV-R2B-σC and rNDV-R2B-FPCS/rNDV-R2B-σC viruses. Moreover, in comparison to the oral swabs, cloacal swabs had shown lesser HA titres in the birds vaccinated with LaSota/rNDV-R2B-σC and rNDV-R2B-FPCS/rNDV-R2B-σC viruses on 2 and 4 dpc (Figure 7A,B).

### 2.8. Pathogenicity of the Recombinant and Virulent Viruses in the Experimental Specific Pathogen Free Chickens

The virus-induced clinical signs and pathological (gross as well as histopathological) changes in the visceral organs were observed after a challenge with virulent NDV and ARV in the experimental birds. The control birds challenged with virulent NDV showed typical symptoms of the disease like inappetence, depression, ruffled feathers, greenish diarrhea, dyspnea, torticollis and succumbed to NDV within 4 dpc. However, the birds vaccinated with LaSota/rNDV-R2B-σC, rNDV-R2B-FPCS/rNDV-R2B-σC and rNDV-R2B-FPCS/rNDV-R2B viruses were completely protected after virulent NDV challenge, indicating 100% immune protective efficacy of the recombinant viruses.

**Figure 8:**
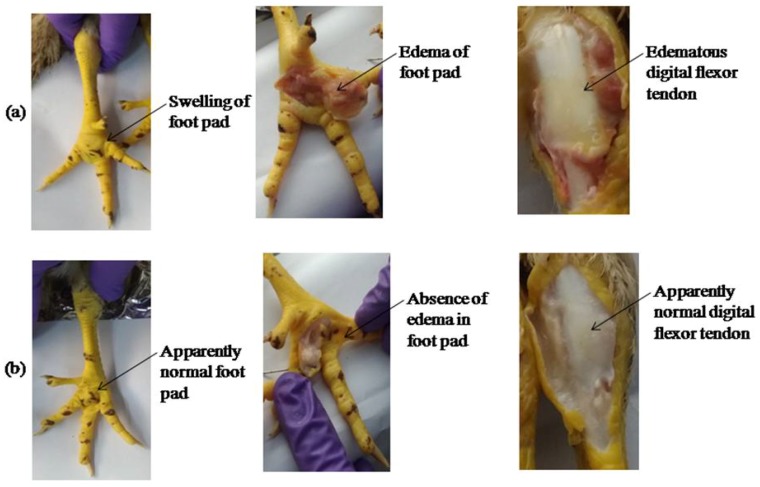
Gross lesions in control and vaccinated birds at 10 days post ARV challenge. (**a**) control birds had swelling and edema of footpads, swollen hock joints with edematous digital flexor tendons; (**b**) vaccinated birds had apparently normal footpads and hock joints.

In the case of a virulent ARV challenge that was carried out using the VA-1 virus isolate, from a chicken suffering from viral arthritis that belonged to the lineage 1 of ARV, the control birds (Figure 8a) showed typical symptoms of ARV infection, such as swelling of hock joint, footpad and diarrhea. However, the birds vaccinated with LaSota/rNDV-R2B-σC, rNDV-R2B-FPCS/rNDV-R2B-σC viruses and ARV inactivated vaccine were apparently normal without any clinical symptoms (Figure 8b).

**Figure 9:**
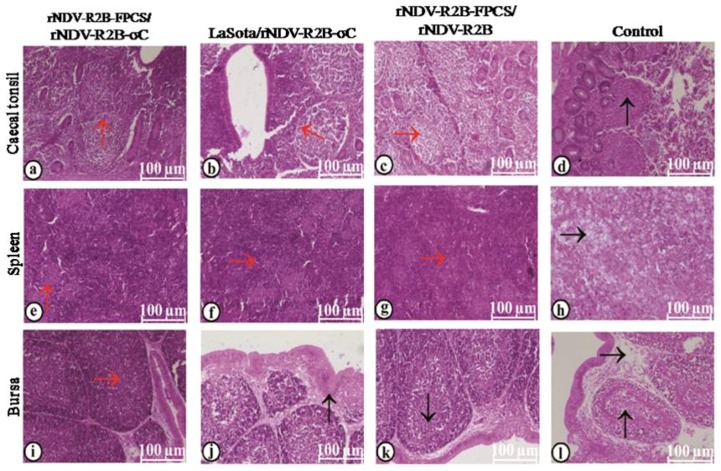
Histopathological changes in various organs following virulent NDV challenge (**a**–**c**) caecal tonsil with normal histoarchitecture; (**d**) caecal tonsil with necrosis and lymphoid depletion; (**e**–**g**) normal histoarchitecture of spleen; (**h**) spleen with follicular necrosis and lymphoid depletion; (**i**) bursa with normal histoarchitecture; (**j**,**k**) section of bursa with mild lymphoid depletion and mild peri-follicular edema; (**l**) bursa with severe lymphoid depletion both in the cortex and medulla with mononuclear infiltration. Scale bar—100 µM. Normal histology of the organs is denoted by red arrows and histopathological changes by black arrows.

Histopathological changes in the visceral organs following virulent virus challenge were studied separately for both NDV and ARV. In the case of virulent NDV challenge, the histopathological changes were studied in the organs like caecal tonsils, spleen and bursa. The birds vaccinated with rNDV-R2B-FPCS/rNDV-R2B-σC (Figure 9a), LaSota/rNDV-R2B-σC (Figure 9b) and rNDV-R2B-FPCS/rNDV-R2B (Figure 9c) viruses showed normal histoarchitecture of lymphoid aggregations in caecal tonsils with intact lining epithelium. However, in the unvaccinated control birds, caecal tonsils revealed severe lymphoid depletion with necrosis of lymphoid follicles and associated reticular cell hyperplasia. The lining epithelial cells were necrotic, and seen to be detached and denuded into the lumen (Figure 9d). The spleen showed normal histoarchitecture with mild lymphoid depletion in the periarteriolar lymphoid sheath (PALS) area in the birds vaccinated with rNDV-R2B-FPCS/rNDV-R2B-σC (Figure 9e), LaSota/rNDV-R2B-σC (Figure 9f) and rNDV-R2B-FPCS/rNDV-R2B (Figure 9g) viruses. In contrast to that, the spleen showed severe lymphoid depletion and associated necrosis of lymphoid follicles in the PALS and lymphoid follicles surrounding the penicillar arteries in the unvaccinated control birds. Moreover, severe hyperplasia of reticular cells in the Schweigger–Seidel sheath (Figure 9h) was noticed. Bursa revealed normal histoarchitecture with intact bursal follicles in cortex and medulla lined by plical epithelium in the birds vaccinated with rNDV-R2B-FPCS/rNDV-R2B-σC viruses (Figure 9i); plical epithelial hyperplasia, mild lymphoid depletion in the medulla and mild perifollicular edema in the group vaccinated with LaSota/rNDV-R2B-σC viruses (Figure 9j); intact plical epithelium, mild lymphoid depletion in the medulla and mild perifollicular edema in group (Figure 9k). However, bursa showed severe plical epithelial hyperplasia, lymphoid depletion both in cortex and medulla, perifollicular edema, mononuclear infiltration and severe necrosis of lymphoid follicles in the medulla, in the unvaccinated control birds (Figure 9l).

**Figure 10:**
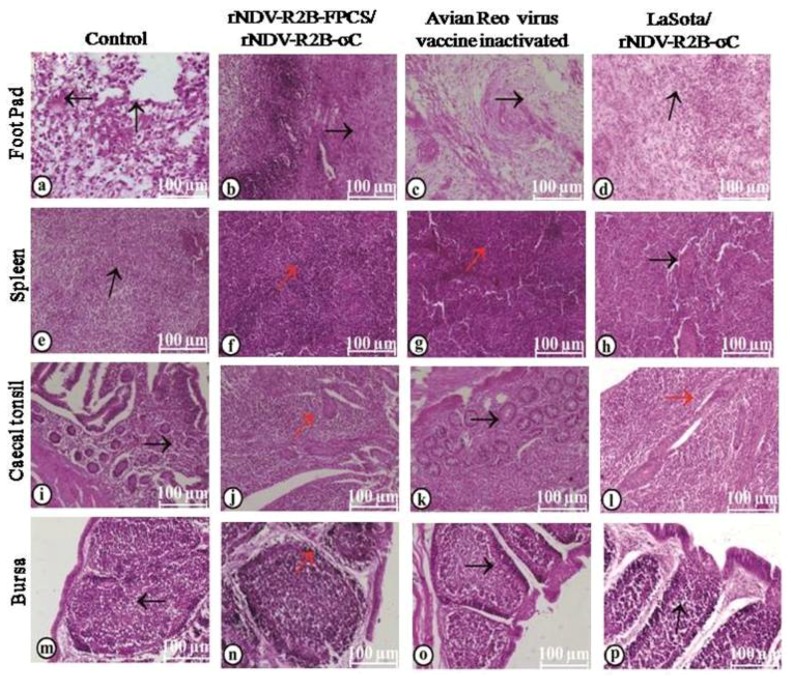
Histopathological changes in various organs following virulent ARV challenge (**a**) the footpad shows hyperkeratosis and necrotic lesions in epidermis and dermis; (**b**,**c**) footpad with mild mononuclear cell infiltration; (**d**) fibrin exudation, edema and mononuclear cell infiltration in the foot pad; (**e**) the spleen shows hyperplasia of reticular cells in Schweigger–Seidel sheath; (**f**,**g**) spleen with normal histoarchitecture; (**h**) spleen with mild lymphoid depletion; (**i**) caecal tonsils show severe lymphoid depletion; (**j**,**l**) caecal tonsils with normal histoarchitecture; (**k**) caecal tonsils show severe necrosis of the lymphoid follicles; (**m**) bursa shows severe lymphoid depletion; (**n**,**p**) bursa shows mild lymphoid depletion; (**o**) bursa shows moderate to severe lymphoid depletion. Scale bar—100 µM. Normal histology of the organs is denoted by red arrows and histopathological changes by black arrows.

Similarly, in the case of virulent ARV challenge, organs like footpad, spleen, caecal tonsils and bursa were collected for histopathological study. The footpad of the unvaccinated control birds showed hyperkeratosis in the epidermal area with necrotic lesions; dermis revealed severe necrosis, fibrin exudation, edema and infiltration of mononuclear cells (Figure 10a). Birds vaccinated with rNDV-R2B-FPCS/rNDV-R2B-σC showed mild hyperkeratosis in the epidermal area of footpad and dermis revealed mild edema, lymphoid follicle formation with mild infiltration of mononuclear cells (Figure 10b). Similarly, mild hyperkeratotic changes in the epidermis along with mild edema and presence of few mononuclear cells in the interstitium of the dermis were seen in the birds vaccinated with an ARV inactivated vaccine (Figure 10c). In the group vaccinated with LaSota/rNDV-R2B-σC, footpad showed fibrin exudation along with edema and severe infiltration of mononuclear cells (Figure 10d). In the unvaccinated control birds, the spleen showed multifocal areas of focal to coalescing lymphoid depletion in both PALS and periellipsoidal lymphoid sheath (PELS). The red pulp area was severely congested and there was hyperplasia of reticular cells in the Schweigger–Seidel sheath (Figure 10e). In contrast, the spleen showed apparently normal histoarchitecture with a normal distribution of lymphoid cell in the PALS and PELS area in the birds vaccinated with rNDV-R2B-FPCS/rNDV-R2B-σC (Figure 10f) and ARV inactivated vaccine (Figure 10g). However, the spleen showed mild lymphoid depletion in the birds vaccinated with LaSota/rNDV-R2B-σC viruses (Figure 10h). The caecal tonsils showed severe lymphoid depletion with intact lining epithelium in the unvaccinated control birds (Figure 10i). However, it showed normal histoarchitecture with lymphoid cell accumulation and follicle formation with intact lining epithelium in the birds vaccinated with rNDV-R2B-FPCS/rNDV-R2B-σC (Figure 10j) and LaSota/rNDV-R2B-σC viruses (Figure 10k). In the birds vaccinated with ARV inactivated vaccine, the caecal tonsils showed lymphoid depletion and severe necrosis of lymphoid follicles in the lamina propria and reticular cell hyperplasia with an incidence of necrosis of enterocytes lining the lymphoid follicles (Figure 10l). Bursa revealed moderate to severe lymphoid depletion both in cortex and medulla with thinning of lymphoid cells in the cortex, vacuolation in the medulla and mild perifollicular edema in the control birds (Figure 10m). In the birds vaccinated with rNDV-R2B-FPCS/rNDV-R2B-σC (Figure 10n) and LaSota/rNDV-R2B-σC viruses (Figure 10o), bursa showed mild lymphoid depletion both in cortex and medulla, thinning of lymphoid cells present in the cortex, mild perifollicular edema and intact plical epithelium. The bursa of the birds receiving the ARV inactivated vaccine showed moderate to severe lymphoid depletion both in cortex and medulla, thinning of the cortex along with hyperplastic plical epithelium (Figure 10p).

## 3. Discussion

In India and other endemic countries, infection with NDV is a perennial problem with outbreaks occurring every year. Similarly, ARV infections are re-emerging under field conditions and cause significant economic losses to the broiler industry as well as breeding stocks. In this context, the development of a bivalent vaccine candidate that can afford significant protection against both of these viral infections gains utmost importance. The present work described here is a seminal work in the development of a vaccine candidate on similar lines using the backbone of an Indian NDV mesogenic vaccine strain R2B [16] and its modified version, with an altered FPCS [35] which can be used in primary vaccination schedule. Biologically, the rNDV-R2B-FPCS virus had an MDT and ICPI values of >168 h and 0.0 indicating a loss in virulence as compared to the parent NDV R2B virus. Furthermore, back passage studies showed the virus to be safe in chickens less than six weeks of age as proven by our earlier studies [35]. Recent outbreaks with ARV field isolates, due to considerable genetic and antigenic variations, are the main causes of vaccination failure [36]. Usually, the breeder flocks are vaccinated with live attenuated vaccine up to 12 weeks of age, followed by 2–3 vaccinations with inactivated oil emulsion before laying. Thus, in endemic countries, it is a common practice towards vaccination of breeder replacement chickens against economically important NDV and ARV. 

The mesogenic NDV vaccine strain R2B that is used as a backbone in this study gives immunity up to one year and is routinely used for booster vaccination in 6–8 week-old birds that are primed with a lentogenic NDV strain. Thus, the primary aim of this study was to develop a bivalent vaccine candidate against NDV and ARV by reverse genetics technology and evaluate its immunogenicity and efficacy in chickens. The σC protein of ARV is the most immunogenic protein of ARV that induces the production of neutralizing antibodies [37]. It can induce the production of antibodies to an extent as produced by the whole virus [38]. In the present study, the ORF of σC gene was amplified from an Indian ARV isolate that belongs to lineage 1 of ARV so as to generate sufficient immune response against circulating ARVs in this geographical location. The σC gene cassette was inserted between the P and M genes of the infectious clone of R2B, as this site for insertion of a gene into the NDV genome was found to be optimal [39]. The rescued recombinant virus, rNDV-R2B-σC showed a slight reduction in growth than the parent virus rNDV-R2B [16]. The MDT and ICPI values indicated that the rescued recombinant virus, although mesogenic in nature, had a slight reduction in virulence due to insertion of the foreign gene which exerts further attenuation of the virus [14].

In this study, both NDV and ARV specific immune responses, as well as the protective efficacy of the rescued recombinant viruses were evaluated and compared with the commercially available live NDV vaccine LaSota and ARV inactivated vaccine. The mesogenic vaccine strain R2B cannot be given to chicks that are less than six weeks old due to the presence of the virulence amino acid motif at the FPCS of the virus. To counteract this, the FPCS region of the virus was altered to generate a recombinant R2B virus that can be used as a primary vaccine [35]. In the present study, this recombinant virus, rNDV-R2B-FPCS, was used as a primary vaccine candidate so that both the primary and booster vaccine candidates were from the same backbone. It is the booster virus vaccine backbone rNDV-R2B that carried and delivered the σC gene of ARV. 

Following vaccination, the recombinant NDV generated an effective humoral immune response against ND and ARV infection. Protection against NDV is primarily by the humoral immune response, as is evidenced by several earlier vaccine studies [17,40,41,42]. Moreover, the anti-σC antibody response generated with the recombinant virus was comparable with the ARV inactivated vaccine. 

The NDV specific stimulation indices obtained at 49 and 56 days of age indicated that the recombinant viruses have more potential to activate the T-cell response in comparison to the commercial live NDV vaccine. Furthermore, the expression level of IFN-γ was the highest among all the cytokines studied, indicating the induction of greater Th1 associated responses against NDV and σC antigens of the recombinant virus. In comparison to the IFN-γ, the IL-4 expression was lower, since IFN-γ helps in the induction of Th-1 responses by inhibiting the Th-2 cytokine production [43]. Protection studies carried on experimental birds showed that the recombinant virus induced 100% protection against a virulent NDV challenge at 62 days of age. In contrast, all the birds from the control group died by 4 dpc. The birds from other vaccinated groups were apparently healthy without any detectable symptoms of NDV infection. In terms of ARV challenge, the birds vaccinated with recombinant rNDV-R2B-σC virus had minimal gross and histopathological lesions as compared to unvaccinated control birds that showed typical histopathological changes in the tissues studied but survived following virulent ARV challenge.

Post-challenge analysis of virus shedding showed that there was a drastic reduction in the shedding of NDV due to the robust immune response generated by the R2B backbone in which the altered FPCS was used for primary vaccination. In earlier studies, researchers have reported that mutations introduced in the FPCS of velogenic and mesogenic strain make these viruses much safer to use, with reduced virus shedding and increased immunogenicity [35,44,45,46,47]. Furthermore, no gross pathological lesions and histopathological changes were observed in the vaccinated birds following virulent NDV challenge. However, marked histopathological changes were observed in caecal tonsils, spleen and bursa of the unvaccinated NDV challenged control birds. Similarly, histopathological alterations were observed in footpad, spleen, caecal tonsils and bursa of the unvaccinated control birds following virulent ARV challenge. In contrast, normal histoarchitecture was observed in the footpad, spleen, caecal tonsils and bursa of the vaccinated birds. 

In summary, we have generated an infectious cDNA clone of NDV strain R2B expressing the σC gene of ARV (pNDV-R2B-σC) and successfully rescued the recombinant NDV-R2B-σC virus by reverse genetics technology. The rescued recombinant virus on biological characterization indicated a reduction in virulence with similar growth characteristics as compared to the parent virus. The recombinant virus on usage as a bivalent vaccine candidate in six6- week-s old chickens induced both humoral and cell mediated immune responses and afforded protection against NDV and ARV infections. This capability gains importance in a scenario wherein ARV vaccination failures occurs in the field due to incompatibility between the circulating strains of the virus and the vaccine virus. The generation of an inactivated vaccine based on the existing viral strain of a geographical area takes much longer time and effort than the present strategy in which the σC gene of the circulating strain of ARV is engineered to be delivered by the NDV vector. 

## 4. Materials and Methods

### 4.1. Ethics Statement

The present study was conducted in strict accordance with the guidelines and recommendations of the Institute Animal Ethics Committee vide F.26-1/2015-16/J.D(R).

### 4.2. Cell Line, Viruses and SPF Eggs

Vero cells were obtained from the American Type Culture Collection (ATCC, Manassas, VA, USA) and were maintained in M199 medium (Life Technologies, Grand Island, USA) supplemented with 10% fetal bovine serum (Hyclone, Pittsburgh, USA). The recombinant NDV strain R2B rescued by reverse genetics was available in the Recombinant DNA Laboratory, Division of Veterinary Biotechnology, Indian Veterinary Research Institute, Bareilly, India. An Indian field isolate of ARV (VA-1) was obtained from the Avian Diseases Section, Indian Veterinary Research Institute [48] and was propagated in primary chicken embryo fibroblast culture. One day-old fertile specific-pathogen-free (SPF) eggs, were purchased from Venky’s India Pvt. Ltd., Pune, India and incubated at 37 °C with a relative humidity of 55% at the Hatchery facility of Central Avian Research Institute, Bareilly, India. Virus propagation and determination of 50% egg infection dose (EID_50_) were done in 9-days-old SPF eggs. NDV vaccine LaSota and ARV inactivated vaccines were purchased from Venky’s India Pvt. Ltd., Pune, India.

### 4.3. Generation of Infectious Clone NDV-R2B Harboring the σC Gene of ARV

The full-length infectious clone of NDV strain R2B (pNDV-R2B) [16], the three support plasmids harboring the nucleoprotein (pNP), phosphoprotein (pP) and large polymerase (pL) genes of NDV and the clone containing the ORF of avian reovirus σC gene (pσC) (Accession number EU681254.1) were available in the Recombinant DNA Laboratory, Indian Veterinary Research Institute, Bareilly, India. The σC gene cassette was generated by PCR containing overlapping fragments of 215 bp (containing NDV P gene end and M gene start signals), 990 bp (ORF of the σC gene) and 499 bp (containing NDV P gene end and M gene start signals) that were amplified with three sets of primers (P1/P2), (P3/P4) and (P5/P6), respectively. Two restriction enzymes sites viz., *Pac*I and *Avr*II were introduced artificially into primers P1 and P6, respectively, for directional cloning. The pNDV-R2B was used as a template for amplification of the fragments using primer pairs P1/P2 and P5/P6 respectively. Similarly, the clone pσC was used as a template to amplify the second fragment by PCR. An additional stop codon (TAA) was placed at the end of the gene to maintain the “rule of six”. The three overlapping fragments were used as a template to assemble the σC gene cassette by PCR with primer pairs P1 and P6. The generated gene cassette was cloned into cloning vector pCR2.1TOPO (Invitrogen, Carlsbard, USA). The recombinant clone was confirmed by restriction enzyme digestion and sequencing and was designated as pCR2.1TOPO-σC. The clone pCR2.1TOPO-σC was digested with restriction enzymes *Pac*I and *Avr*II and the resulting insert was sub-cloned into pNDV-R2B-FL between the intergenic region of P and M genes at nucleotide position 3077 and 3568. The resulting recombinant clone was confirmed by restriction enzyme digestion as well as by sequencing and was designated as pNDV-R2B-σC. The recombinant NDV harbouring the σC gene was rescued by reverse genetics techniques [16]. Briefly, Vero cells were grown up to 80% confluence and transfected with the recombinant clone pNDV-R2B-σC along with three support plasmids as per manufacturer’s instruction (Invitrogen, USA). Before transfection, the culture medium was removed from the cells and the cells were incubated with OptiMEM for 1 h at 37 °C in a CO_2_ incubator. The recombinant plasmid pNDV-R2B-σC (5 µg) together with support plasmids (2.5 µg pNP, 1.5 µg pP and 0.5 µg pL) were diluted in 500 µl of the OptiMEM medium containing Lipofectamine 3000 reagent (Invitrogen, Carlsbard, USA) and incubated at 37°C for 30 min. Following the incubation period, OptiMEM media was removed and the lipofectamine-plasmid mixture was added gently onto the cells. One mL of OptiMEM media was added to the cells and the cells were incubated at 37 °C for 24 h. The transfection mixture was removed after 24 h of incubation and M199 medium (Life Technologies, Grand Island, USA) was added to the cells and was left undisturbed for 3 days. Following the incubation period, cells were freeze-thawed three times and the culture supernatant was used for re-infection of healthy confluent Vero cells. Monolayers of the cells were observed for the development of cytopathic effect under the inverted microscope (Leica Microsystems, Wetzlar, Germany) and the same process was repeated for 10 times. After 10 passages in Vero cells, the culture supernatant was inoculated into 9–11 days old embryonated SPF chicken eggs and incubated for 72 h at 37 °C. After completion of the incubation period, allantoic fluid was harvested aseptically and tested by haemagglutination (HA) test to confirm the presence of the recombinant NDV. The total RNA was isolated from the infective allantoic fluid and subjected to reverse transcription PCR (RT-PCR) to amplify σC gene using the primer pairs P3 and P4. The resultant recombinant virus was designated as rNDV-R2B-σC.

### 4.4. Biological and Molecular Characterization of the Recombinant Virus rNDV-R2B-σC 

The recombinant virus was propagated in 9–11 days old embryonated SPF chicken eggs for bulk purification. In addition, 100 mL of allantoic fluid from the 10th egg passage was harvested and the virus was purified by ultracentrifugation by established protocols [16]. Briefly, the virus pellet obtained by ultra centrifugation of allantoic fluid done at 35,000× *g* for 2.5 h was purified on a sucrose gradient consisting of 55% and 20% sucrose and centrifuged at 45,000× *g* for 1 h.

#### 4.4.1. Immunofluorescence Assay (IFA)

A confluent monolayer of Vero cells was infected with the recombinant virus rNDV-R2B-σC at a multiplicity of infection (MOI) of 0.01 and incubated at 37 °C. After 72 h of incubation, both the infected and uninfected monolayers were washed with PBS and fixed with 4% paraformaldehyde (Affymetrix Inc., Cleveland, USA) for 90 min at 37 °C. Following the incubation period, cells were treated with 0.5% Triton X-100 (Sigma, St.Louis, USA) for 10 min at room temperature to permeabilize the membrane. Permeabilized cells were blocked with 5% bovine serum albumin (Sigma, St.Louis, USA) for 30 min at room temperature. Cells were washed three times with PBS and incubated for 1 h with a mixture of antibodies. Briefly, cells were incubated for 1 h with a mixture of anti-NDV polyclonal antibody (Abcam, Cambridge, UK; 1:50 dilution) and ARV specific primary antibody (Abcam, Cambridge, UK; 1:100 dilution). Following the incubation period, cells were washed three times with PBS and incubated with a mixture of Alexa Fluor (R) 568-labelled goat anti-chicken IgG (Molecular Probes, Eugene, USA, 1:200 dilution) and FITC labelled rabbit anti-chicken IgG (Sigma, USA; 1:250 dilution) and kept for 1 h at room temperature. Nuclear staining was performed using DAPI (Molecular Probes, Eugene, USA) at 1:1000 dilution. Finally, the cells were visualized for corresponding fluorescence and images were taken using Fluo View FV1000 confocal microscope (Olympus, Tokyo, Japan) at the matching excitation and emission filters for FITC and Alexa Fluor 568.

#### 4.4.2. Growth Kinetics of the Recombinant Viruses

The growth pattern of the recombinant viruses rNDV-R2B-σC and rNDV-R2B were evaluated by multiple-cycle of growth conditions. Monolayers of Vero cells in six well plates were infected with the recombinant virus at an MOI of 0.01. Cells were incubated at 37 °C for 1 h for adsorption of the virus and culture media was replaced after 1 h with fresh M199 medium and kept for incubation at 37 °C. The culture supernatant was collected at every 12 h interval up to a period of 72 h and the virus titre in the harvested culture supernatant was determined by TCID_50_ method. Briefly, a ninety-six well plate with confluent Vero cells (80%) was infected with a serial 10-fold dilution of the recombinant virus present in the infective culture supernatant. The plate was incubated at 37 °C for 1 h for adsorption of the virus. After completion of the adsorption period, medium M-199 was added to the wells and incubated at 37 °C for 3 days. Cells were observed under a microscope for the appearance of cytopathic effect and virus titres were calculated using the standard method [49].

### 4.5. Pathogenicity Evaluation

The pathogenic attributes of the recombinant viruses rNDV-R2B-σC and rNDV-R2B were evaluated by mean death time (MDT) and intra-cerebral pathogenicity index (ICPI) analysis as per standard methods [50]. To evaluate the stability of the recombinant virus, it was serially passaged for 10 times in 9-day-old embryonated SPF chicken eggs. Allantoic fluid was harvested from the 10^th^ egg passage and was used for total RNA isolation using TRIzol reagent (Invitrogen, Carlsbad, USA). Extracted RNA was subjected to RT-PCR to check the presence of a σC gene cassette.

### 4.6. Titration of the Recombinant Virus

The concentration of the recombinant virus rNDV-R2B-σC was expressed as an infectivity titre and it was done by carrying out a titration. The infectivity titre of the recombinant virus was expressed as 50% embryo infectious dose (EID_50_)_._ For that, a serial 10-fold dilution of the recombinant virus was made in normal saline solution and five 11-day-old SPF embryonated chicken eggs were inoculated with each dilution and incubated for 4 days. Following the incubation period, allantoic fluid was harvested and HA test was performed to determine the presence or absence of the virus. The infectivity titre of the virus was calculated using the standard method [49].

### 4.7. Immunogenicity and Protective Efficacy of the Recombinant NDV Expressing the σC Gene of ARV

The immunogenicity and protective efficacy of the recombinant virus rNDV-R2B-σC was evaluated in SPF chickens. A total of 48 chickens were randomly divided into 5 groups (Table 1). Pre-challenge serum samples were collected at weekly intervals from all the birds. Humoral immune response was evaluated by haemagglutination inhibition (HI) test and recombinant nucleocapsid protein-based enzyme-linked immunosorbent assay (ELISA) for checking the antibodies against NDV and recombinant σC protein-based ELISA for checking the anti-σC antibody of ARV. Cell-mediated immune response was evaluated by antigen specific lymphocyte transformation test and gene expression analysis of cytokine genes IL–1β, IL–4, IL–12, IL–13 and IFN-γ using real-time PCR (Bio-Rad, USA) with published primers [51].

To determine the protective efficacy of the recombinant viruses, birds (n = 6) from groups one, two, three and five were challenged with virulent NDV (10^5^ ELD_50_/mL/bird) and birds (n = 6) from group one, three, four and five were challenged with virulent ARV (10^5^ TCID_50_/mL/bird) [48] at 62 days of age. Following the challenge, birds were observed for 10 days to study for the appearance of clinical signs of both NDV and ARV infection and the surviving birds were sacrificed humanely. Following the challenge, oropharyngeal and cloacal swabs were collected aseptically in brain heart infusion broth (BD Diagnostics, Gurgaon, India) from the birds on 2- and 4-day post-challenge (dpc) to determine the shedding of the virus. The swab samples collected from the same group were pooled together and clarified by centrifugation at 1000× *g* for 15 min and was inoculated in 9-day- old embryonated SPF chicken egg through allantoic cavity route. After 72 h of incubation of the eggs at 37 °C, allantoic fluid was harvested and used in the HA assay to check the presence of the virus as well as to determine the viral titre.

To study the histopathological changes due to virulent NDV and ARV challenges, organs viz., spleen, bursa of Fabricius, caecal tonsils, footpad were collected from the experimental birds and fixed in 10% neutral buffered formalin (NFB). The formalin-fixed organ samples were subjected to processing by paraffin-embedded sectioning and 4–6 µm sections were made and mounted onto the slides for haematoxylin and eosin (H&E) staining. Stained slides were viewed under an inverted light microscope (Leica Microsystems, Wetzlar, Germany) for detection of histopathological changes.

## 5. Conclusions 

The present work generated a recombinant NDV incorporating σC gene of ARV by reverse genetics to be used as a bivalent vaccine candidate that affords 100% protection against NDV and ARV infections in SPF chickens. This can help the poultry industry in reducing the number of vaccines given to birds and the cost of vaccination to a greater extent. However, the efficacy of this vaccine needs to be explored further in young chickens harbouring maternal antibodies.

## Figures and Tables

**Table 1:** Immunization protocol for evaluation of the protective efficacy of the recombinant virus as a vaccine.

Groups	Vaccination		Age of Vaccination (Day)		Dose		Route	
	Primary	Booster	Primary	Booster	Primary	Booster	Primary	Booster
1	rNDV-R2B-FPCS	rNDV-R2B-σC	7	42	100 µL 10^6^ EID_50_/mL	100 µL 10^6^ EID_50_/mL	IO/IN	IM
2	rNDV-R2B-FPCS	rNDV-R2B	7	42	100 µL 10^6^ EID_50_/mL	100 µL 10^6^ EID_50_/mL	IO/IN	IM
3	LaSota	rNDV-R2B-σC	7	42	100 µL 10^6^ EID_50_/mL	100 µL 10^6^ EID_50_/mL	IO/IN	IM
4	Avian Reovirus vaccine inactivated		42	-	0.5 mL 10^6^ TCID_50_/mL	-	IM	-
5	Control (PBS)	Control (PBS)	7	42	100 µL	100 µL	IO/IN	IM

IO: intra oral; IN: intra nasal; IM: intra muscular.
